# *In vitro* differentiation of germ cells from stem cells: a comparison between primordial germ cells and *in vitro* derived primordial germ cell-like cells

**DOI:** 10.1038/cddis.2015.265

**Published:** 2015-10-15

**Authors:** W Ge, C Chen, M De Felici, W Shen

**Affiliations:** 1 Institute of Reproductive Sciences, Qingdao Agricultural University, Qingdao 266109, China; 2 Key Laboratory of Animal Reproduction and Germplasm Enhancement in Universities of Shandong, College of Animal Science and Technology, Qingdao Agricultural University, Qingdao 266109, China; 3 Department of Biomedicine and Prevention, University of Rome ‘Tor Vergata', Rome 00133, Italy

## Abstract

Stem cells are unique cell types capable to proliferate, some of them indefinitely, while maintaining the ability to differentiate into a few or any cell lineages. In 2003, a group headed by Hans R. Schöler reported that oocyte-like cells could be produced from mouse embryonic stem (ES) cells *in vitro*. After more than 10 years, where have these researches reached? Which are the major successes achieved and the problems still remaining to be solved? Although during the last years, many reviews have been published about these topics, in the present work, we will focus on an aspect that has been little considered so far, namely a strict comparison between the *in vitro* and *in vivo* developmental capabilities of the primordial germ cells (PGCs) isolated from the embryo and the PGC-like cells (PGC-LCs) produced *in vitro* from different types of stem cells in the mouse, the species in which most investigation has been carried out. Actually, the formation and differentiation of PGCs are crucial for both male and female gametogenesis, and the faithful production of PGCs *in vitro* represents the basis for obtaining functional germ cells.

## Facts

Some stages of gametogenesis occurring in the embryo or foetus have been reproduced and significant progress in obtaining mature oocytes and spermatozoa from postnatal gonads *in vitro* were already achieved.
The capability of stem cell-derived PGC-LCs to give rise to functional gametes has been investigated in a few papers with partial positive results.
The artificial germ cells produced from mouse pluripotent stem cells proved to be functional as they were capable to differentiate into spermatozoa and oocytes that can give rise to live progeny.

## Open Questions

What are the main differences between embryo- or foetus-derived PGCs and stem cell-derived PGC-LCs?
Whether artificial germ cells can be utilized for medical purpose for human in the future?
Are those viable progeny produced from stem cell-derived gametes true healthy individuals?
Whether *in vitro* conditions are sufficient for PGC-LCs entering into meiosis and completing epigenetic reprogramming?

From the first work by Hübner *et al.* in 2003,^1^ showing that oocyte-like cells (OLCs) could be produced from mouse embryonic stem (ES) cells *in vitro*, derivation of germ cells from stem cells raised great public sensation, inspiring hopes in some, fears in others.^2, 3, 4, 5, 6, 7^ The Hübner's study and those that followed definitively dispelled the already unsteady myth of the unique and mysterious characteristics of the germline. Actually, in mammals, germ cells derive from the mortal soma and non-continuous germline do exist. Many researchers devoting to the germ cell biology were, however, surprised by some of these results. In fact, artificial germ cells produced in the Hübner's paper and in others that rapidly followed uncovered *in vitro* developmental capabilities that were not shown by true germ cells. Oocytes and sperm seemed to magically appear in the culture dish, although scientists of reproductive biology know that gametogenesis is a very complex process of which only some stages can be reconstructed *in vitro*. At the same time, however, they were fascinated by these results for two main reasons. First, once validated, *in vitro* model of gametogenesis from stem cells could make it easier to study and elucidate the mechanisms underlying gametogenesis, mainly in the humans, in which experimental approaches are limited. Second, artificial germ cells could greatly improve and make the actual procedures of assisted reproductive technology more efficient and develop alternative infertility treatments. A scenario for radical changes in the reproduction performance of many species, first of all humans, could also be imagined with consequences difficult to foresee. Actually, from Hübner's work, many papers described the production of germ cells from various types of stem cells, even includes humans.^8^ Particularly important, some authors reported the generation of live offspring from male and female germ cell-like cells obtained *in vitro* from mouse ES and induced pluripotent stem (iPS) cells.^9, 10^ As both male and female germline begins from primordial germ cell (PGCs), precise information about the characteristics and developmental capability of the embryo-derived PGCs and their counterpart, PGC-like cells (PGC-LCs) produced *in vitro* from stem cells, is essential to elucidate the conditions for obtaining functional germ cells. The present review is focused on this topic.

## Brief Outline of Mouse Gametogenesis

In the attempt to produce germ cells or recreate gametogenesis phases *in vitro*, it is essential to establish the type and the developmental stage of the putative germ cell generated. As most of the studies reviewed here have been performed in the mouse, we summarized a brief outline of murine gametogenesis and key genes involved in germ cell formation and their roles during gametogenesis, as shown in Table 1.

In the mouse embryo, PGC precursors arise around 6.25 dpc from the proximal epiblast. Subsequently, the precursors move into the extraembryonic allantois mesoderm where they are specified as PGCs. From 8.0 dpc, PGCs migrate into the embryo proper, moving through the hindgut and the dorsal mesentery and around 10.0 dpc begin to colonize the gonadal ridges. Germ cell proliferation occurs throughout the migratory period, and continues for 2–3 days after gonad entering, forming a population of about 25 000 cells at 13.5 dpc when proliferation terminates.^11^ As many mitoses do not end with cytokinesis, germ cells give rise to small cell clusters termed cysts or nests. Subsequently, they either cease mitosis and enter meiosis becoming primary oocytes (dictyate or germinal vesicle stage) in the female or progressively arrest in G0/G1 as prospermatogonia/gonocytes in the male.

In the female, around birth, oocytes arrest at the diplotene stage of the meiotic prophase I, and undergo a loss of more than one-third of their number by apoptosis and/or autophagy.^12, 13^ Subsequently, the surviving oocytes are individually encircled by pregranulosa cells and finally enclosed into a primordial follicle. The primordial follicle pool represents the ovarian reserve from which cohorts of follicles are continuously activated from birth to menopause to undergo extensive growth and development as primary and secondary follicles in a process termed folliculogenesis.

In the male, transformation of gonocytes into SSCs occurs between 0 and 6 dpp, with the first appearance of biologically active SSCs occurring at approximately 3–4 dpp.^14^ The first round of spermatogenesis is completed by puberty and continues up to 1–2 years of age.^15^ The seminiferous cords of the testis of an adult mouse contain cell types in three principal phases of spermatogenesis lasting as whole for 33–35 days:^16, 17^ spermatogonial renewal and proliferation, meiosis, and spermiogenesis.

## *In Vitro* and *In Vivo* Gametogenesis from Embryo-Derived PGCs

The process of female or male gametogenesis from the formation of PGCs to functional oocytes or sperm has not been entirely recreated *in vitro* in any mammalian species. However, some stages of this process occurring in the embryo or foetus have been reproduced and significant progresses in obtaining mature gametes from postnatal gonads *in vitro* were achieved. At present, the more promising approaches for producing functional gametes from PGCs are based on *in vivo* transplantation of PGC-containing tissues collected from embryos or after *in vitro* reaggregation of PGCs with somatic gonadal cells, into the gonads of prepuberal/adult hosts.

### *In vitro* derivation of PGCs from epiblast

In 2005, Ohinata *et al.*^18^ devised a robust *in vitro* culture protocol to induce the differentiation of epiblast cells into PGC-LCs. They added BMPs and WNT3 to the culture dish of isolated floating epiblasts and monitored PGC formation using transgenic fluorescent reporter genes whose expression is controlled by the upstream regulatory elements of the genes encoding *Prdm1* (also known as *Blimp1*) or *Prdm14*, which are expressed in PGC precursors.^18, 19, 20, 21^ They showed that epiblast cells, in which these reporters were induced, expressed germ cell-specific genes in an appropriate sequence and displayed chromatin modifications characteristic resembling *in vivo* germ cells. Most importantly, they also demonstrated that male PGC-LCs, like endogenous PGCs, were able to differentiate into spermatozoa when transplanted into testicular tubules of prepuberal mice and even to fertilize oocytes to produce viable mice.

### Oocytes and EG cells from *in vitro* cultured and isolated PGCs

**Figure 1:**
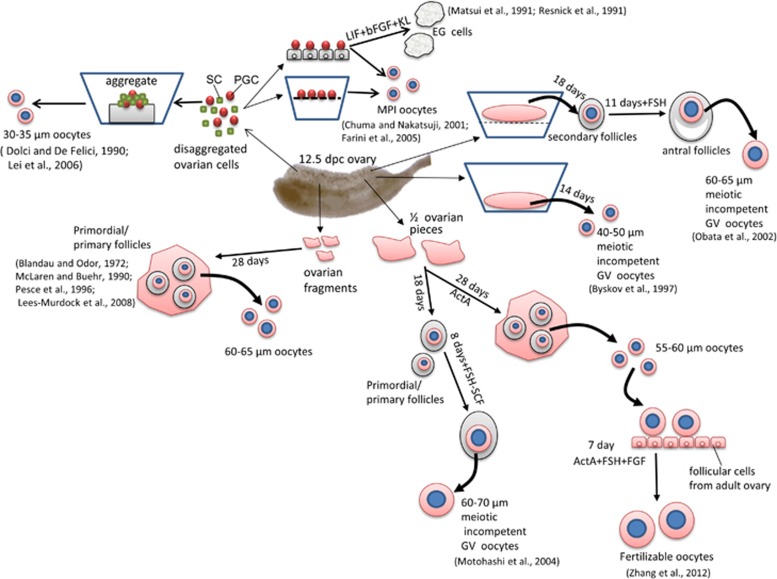
A schematic representation of the main experimental approaches and results used to reproduce *in vitro* oogenesis stages from premeiotic female mouse PGCs (for details, see text)

**Figure 2:**
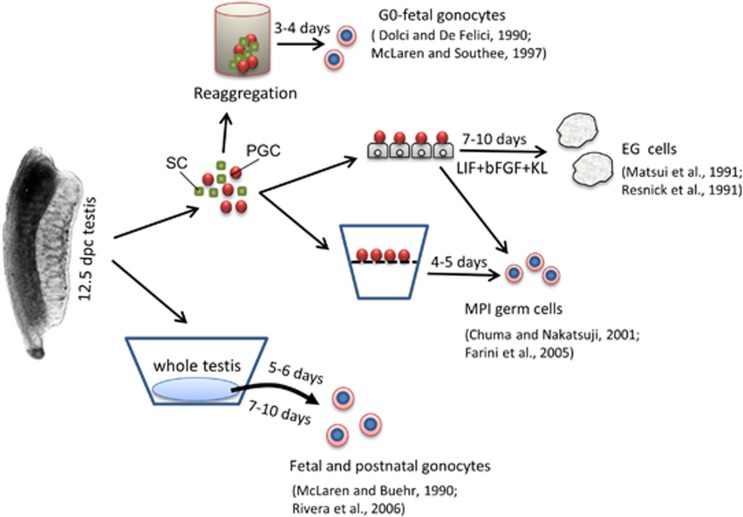
A schematic representation of the main experimental approaches and results used to reproduce *in vitro* spermatogenesis stages from 12.5-dpc male mouse PGCs (for details, see text)

Usually mouse PGCs obtained from 11.5 to 12.5-dpc gonadal ridges can be maintained in culture only for 3–4 days before undergoing degeneration through apoptosis (Figures 1 and 2).^22, 23^ Although the cell monolayers were considered necessary to support PGC survival and proliferation over this time, Farini and her collaborators^24^ showed that they were not necessary for their entering into meiosis *in vitro*. Actually, these authors showed that both female and male PGCs with minimal gonadal cell contamination were able to enter and progress into meiotic prophase I up to pachytene/diplotene when providing a cocktail of growth factors. On the other hand, it has been previously shown that mouse PGCs maintained onto embryonic fibroblast monolayers for 7–10 days in the continuous presence of a combination of growth factors, deviated from their normal differentiation pathways and gave rise to colonies of ES-like cells termed EG cells.^25, 26, 27^ Such behaviour was much more frequent in pregonadal (8.5–10.5 dpc=about 18/100) than in gonadal (11.5–12.5 dpc=about 3/100) PGCs and resembled the transformation of PGCs in embryonic carcinoma cells occurring in the foetal testis of certain mouse strains.^28^ Similarly, EG cells were derived from human PGCs.^29^ More recently, it has been shown that mouse PGCs can also give rise to EG cells in the absence of cell monolayers when they were cultured onto fibronectin and in the presence of GSK3 and ERK kinase inhibitors (2i).^30, 31^

As far as we know, only one paper has shown that pregonadal male or female PGCs mixed with endogenous somatic cells of the 10.5 dpc AGM region when cultured onto KL-producing cell monolayer were able to enter and progress into meiotic prophase I following a timing comparable with the *in vivo* situation.^32^ In partial disagreement with such results, Farini *et al.* reported that although isolated 10.5 dpc PGCs were able to engage meiosis and express meiotic genes such as *Sycp3*, *Dmc1* and *Rec8*, these PGCs appeared unable to progress beyond the leptotene stage after 5 days of culture. PGCs of the same age, however, were able to enter and progress into the meiotic prophase I if cultured within an intact AGM.

The capability of the primary oocytes produced *in vitro* from female PGCs to continue their development and be enclosed into follicles was not studied. Indirect data indicate, however, that they could be able to begin the growing phase *in vitro* also without the companion pregranulosa cells but not to form follicles with ovarian somatic cells. In fact, Klinger and De Felici^33^ showed that primary oocytes isolated from the foetal ovaries entered the growth phase upon stimulation with KL, while Lei *et al.*^34^ observed that 12.5-dpc PGCs after 10–11 days of culture developed into primary oocytes, but were unable to form follicles when aggregated with gonadal somatic cells.

Overall, these experiments indicate that both isolated female and male gonadal PGCs *in vitro* are able to enter and progress into meiotic prophase I when the right cocktail of growth factors is provided. In any case, the microenvironment of the developing gonadal ridges seems necessary for gonadal PGCs acquiring meiotic competence and capability to enter and progress into the meiotic prophase I.

### Oocytes and gonocytes from *in vitro* cultured PGCs inside gonadal tissues

The developmental capability of female PGCs maintained *in vitro* within their gonadal tissues was investigated following basically three experimental approaches: organotypic culture, culture of pieces of ovarian tissues or PGC-somatic cell aggregates (Figures 1 and 2).

As far as we know, the more advanced developmental phases of oogenesis obtained using organotypic *in vitro* culture of mouse embryonic ovaries were the preantral and antral follicle stage.^35, 36, 37^ In particular, Obata and collaborators obtained follicles from 12.5 dpc ovaries via a two-step *in vitro* culture system.^37^ Noteworthy, Germinal vesicle oocytes obtained from such follicles reached a diameter of 60–65 *μ*m but were unable to resume meiosis.

Several authors cultured whole or pieces of embryonic ovaries allowing them to attach and spread onto the bottom of the culture dish.^38, 39, 40, 41, 42^ In such cultures, mouse PGCs were able to efficiently form primary oocytes capable to be assembled into primordial or primary follicles. Within these structures, oocytes grew considerably to reach a diameter around 65–70 *μ*m, but did not acquire the capability to resume and complete meiosis as normally do their *in vivo* counterparts. Recently, Zhang *et al.*^42^ reported, however, that a small number of such oocytes, when ActA was present in the medium throughout the entire culture time, were able to resume meiosis and be fertilized.

Finally, in a third experimental approach, female PGCs isolated and aggregated with gonadal cells of the same or other ages or different cell types, were found to enter meiosis and give rise to primary oocytes of 30–35 *μ*m diameter. These cells were, however, unable to form follicles or to further progress into the growing phase *in vitro*.^34, 38, 43^

These *in vitro* experiments emphasize that it is relatively simple to induce the development of female PGCs into primary oocytes inside cultured ovarian tissues *in vitro*. Moreover, they indicate that the most part of these oocytes are capable to complete the meiotic prophase I and to begin and almost complete the growing phase independently from proper folliculogenesis. However, under the *in vitro* conditions devised so far, even the oocytes reaching full growth rarely acquire complete meiotic maturation and fertilizability and never the capacity to support embryo developmental after fertilization.

Little information is available about the developmental capability of male PGCs *in vitro* cultured within embryonic testis or testicular tissues. Early papers showed that 12.5-dpc male PGCs aggregated with their own or different type of somatic cells entered G0 arrest as prospermatogonia as they normally do *in vivo*.^38, 43^ On the other hand, male PGCs obtained from 11.5-dpc gonadal ridges entered into meiosis when aggregated with their own gonadal cells or lung cells.^44^ Similarly, PGCs cultured within intact 12.5-dpc testis underwent G0 arrest but entered meiosis in 11.5-dpc gonadal ridges if testis cords do not properly form.^38, 45^ Once formed, prospermatogonia are able to survive for several days in organotypic cultures and to reach developmental stages comparable with postnatal gonocytes.^46^ However, whether male PGCs from such culture conditions are capable of spermatogenesis remains unknown.

Actually, it was also proven complicated to reconstruct the entire mammalian spermatogenesis *in vitro* from postnatal spermatogonia. Interestingly, new methods have been only recently published reporting complete spermatogenesis *in vitro* from postnatal or prepuberal mouse gonocytes/spermatogonia. Sato *et al.*^47^ showed that tissue fragments of neonatal mouse testes, which contain only gonocytes or primitive spermatogonia, can produce spermatids and sperm *in vitro* under serum-free culture conditions. The obtained spermatids and spermatozoa resulted in healthy and reproductively competent offspring through intracytoplasmic fertilization. Using the same method, these authors reported spermatogenesis also from SSCs.^48^ Another method based on three-dimensional testicular cell culture in soft agar has been recently described to allow the progression of prepuberal spermatogonia into meiosis and further maturation of the germ cells into morphologically normal spermatozoa, although with very low efficiency.^49^

These last results suggest that once and if male PGCs are induced to differentiate into postnatal gonocytes/spermatogonia, complete spermatogenesis could be accomplished *in vitro* using such methods.

### Oocytes and sperm from *in vivo* transplanted PGCs

**Figure 3:**
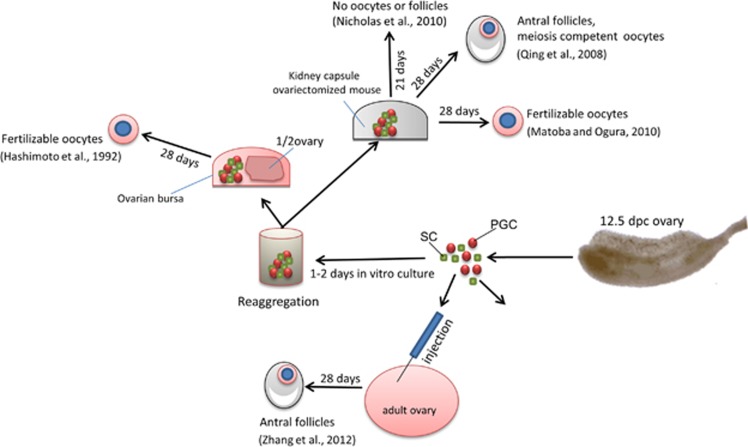
A schematic representation of the main experimental approaches and results used to reproduce *in vivo* oogenesis stages from premeiotic female mouse PGCs (for details, see text)

A number of papers showed that ovaries reconstructed by aggregating premeiotic female PGCs with ovarian somatic cells and transplanted into the kidney capsule or the ovarian bursa of syngeneic hosts are able to form preantral and antral follicles and develop into meiotic competent and even fertilizable oocytes (Figure 3).^34, 50, 51^ In this regard, it is important to point out that the developmental stage of the ovary from which PGCs are isolated and the proper synchronization of the germ cell-somatic cell interactions are crucial for germ cell survival and completion of oogenesis after transplantation. In fact, Nicholas *et al.*^50^ found that in transplanted reconstructed ovaries before 13.5 dpc, PGCs completed prophase I of meiosis but did not survive and form follicles. The same results were obtained when 12.5-dpc PGCs were aggregated with somatic cells from foetal ovaries of later developmental stages, and these PGCs are competent to form follicles after kidney capsule transplantation or *in vitro* culture.^34, 50^ However, other papers cited above^52, 53^ reported that aggregates of 12.5-dpc ovarian cells were able to produce fertilizable oocytes.

More recently, Zhang *et al.*^54^ injected fluorescent GFP-ovarian cells obtained from 12.5-dpc foetuses directly into the ovaries of adult recipient females. After 2 months, they found a significant number of preantral and antral follicles showing both GFP-positive oocyte and surrounding granulosa cells. Zou *et al.*^55^ reported that putative mouse OSCs can produce live offspring when transplanted into the ovaries of adult females. Similar results were also observed in human when putative OSCs isolated from reproductive-age women were injected into adult human ovarian cortical tissue biopsies and further transplanted into immunodepressed female mice.^56^ These results demonstrated that not only the ovary environment is obviously crucial for supporting proper oogenesis but also that oogenesis processes normally occurring during the foetal and early postnatal ages can occur in the postnatal ovary or even in ectopic sites in adult individuals.^57, 58, 59^

**Figure 4:**
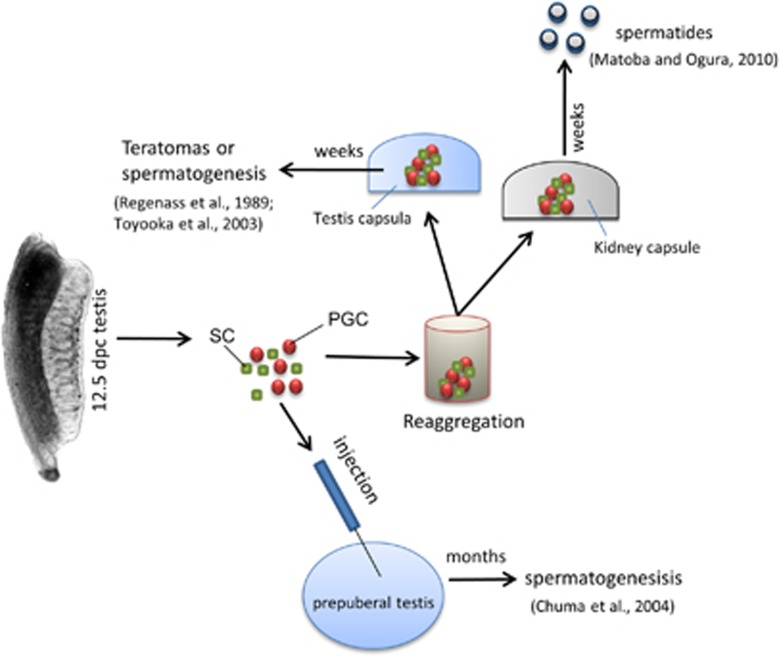
A schematic representation of the main experimental approaches and results used to reproduce *in vivo* spermatogenesis stages from 12.5-dpc male mouse PGCs (for details, see text)

The capability of pregonadal and gonadal male PGCs to undergo spermatogenesis upon *in vivo* transplantation was investigated in a few papers (Figure 4). Previous works showed that 12.5–15.5-dpc mouse testes were capable to reorganize into testicular structures with spermatogenesis or seminiferous tubules with teratomas when transplanted under the capsule of adult testes.^60, 61^ Following the seminal work of Brinster *et al.*,^62^ which showed that the transplantation of spermatogonia from donor mice to the testes of infertile recipient mice results in donor-derived spermatogenesis. Chuma *et al.*^63^ reported that male epiblast cells and PGCs were surprisingly able to establish colonies of spermatogenesis after transfer into the seminiferous tubules of infertile prepuberal mice. Matoba and Ogura^53^ reported that spermatids can be obtained from 12.5-dpc male PGCs when transplanted under kidney capsules of adult mice; significantly, these spermatids can give rise to live offspring when injected into normal oocytes.

Although the development of male PGCs transplanted into adult testes into teratomas or differentiating germ cells seems critically affected by the donor genotype, the findings reported above unequivocally indicate that they may exhibit remarkable spatial and temporal flexibility in development, as long as they remain under *in vivo* conditions. Particularly important, the postnatal testis tubules, at least up to puberty, appear able to provide the male PGCs the proper microenvironment to accomplish their complete maturation process from the epiblast to postnatal spermatogonial stem cells.

## *In Vitro* and *In Vivo* Gametogenesis from PGC-Like Cells

**Figure 5:**
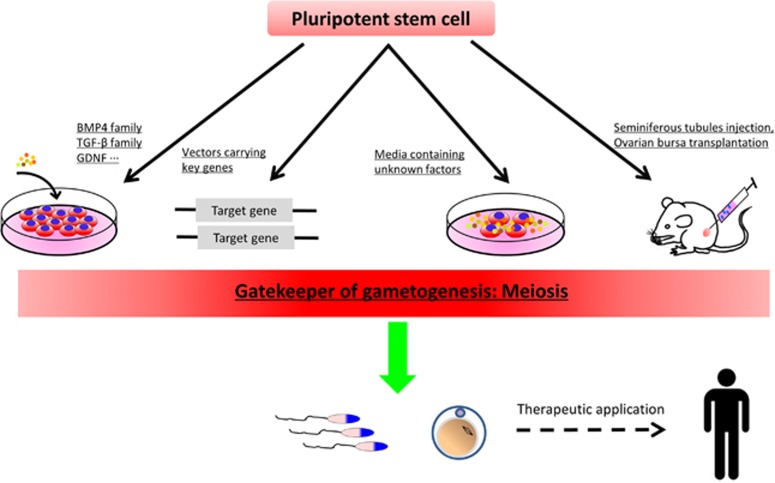
Methods used for germ cell induction and potential medical application

From the ground-breaking work of Hübner *et al.*^1^ until that of Hayashi *et al.*^9, 10^ several papers claimed to have derived PGC-like cells from different types of stem cells ranging from ES to iPS cells and adult stem cells. A detailed description and discussion of these papers is beyond the scope of the present work and can be found elsewhere.^2, 4, 5, 6, 64, 65, 66, 67, 68, 69, 70^ Briefly, the PGC induction methods frequently include: (i) The formation of EBs and the use of growth factors known to induce PGC differentiation from the epiblast such as BMP4 and WNT3 (in some studies, PGC-LCs appeared to form spontaneously from EBs, see for example Geijsen *et al.*,^71^ Vincent *et al.*^72^); (iii) overexpression of key genes that regulate germ cell formation, such as *Dazl* and *Mvh* genes;^73, 74^ and (iii) conditioned media containing unidentified PGC-LC0inducing factors (Figure 5).^75, 76^ Most of these studies had, however, some or several drawbacks. For example, the lack of markers that unequivocally allow to distinguish PGCs from stem cells and the precise molecular characterization of the PGC-LCs produced *in vitro*. Most importantly, as we will discuss in the next paragraphs, the capability of these PGC-LCs to give rise to functional gametes was investigated in a few papers with partial positive results only.

Between 2011 and 2012, the papers by Hayashi *et al.*^9, 10^ established a protocol that can be so far considered the golden standard for derivation of PGCs from ES and iPS cells. In this procedure, stem cells were first differentiated into a novel type of cells harbouring the post-implantation epiblast status, called epiblast-like cells (EpiLCs); after induction with a cocktail of other growth factors, EpiLCs robustly (about 30%) differentiated into PGC-LCs resembling their *in vivo* counterparts. Using a similar method, PGC-LCs have been recently obtained from human ES cells.^8^ Interestingly, the Saitou's group also reported that without growth factors, simultaneous overexpression of three transcription factors, *Blimp1*, *Prdm14* and *Tfap2c* (also known as *AP2γ*), directed mouse EpiLCs swiftly and efficiently into a PGC state.^77^ An apparent improvement of the original Hayashi's method has been recently reported.^78^ As we will discuss below, Hayashi *et al.* notably reported that PGC-LCs were capable to differentiate into fertile sperm and oocytes after *in vivo* transplantation.

### Oocytes and male germ cells from isolated PGC-like cells *in vitro*

As far as we know, no papers have convincingly shown that PGC-LCs are able to undergo normal meiotic prophase *in vitro*. In fact, although several authors reported the expression of meiotic genes and proteins by these cells such as SCP3, *γ*H2AX or DMC1, in a few of them, in which such capability was investigated by observing the typical morphological pattern of the meiotic chromosomes, severe abnormalities were reported.^79, 80, 81^

Despite such results, in the early Hübner's study and in a number of subsequent papers, the formation of follicle-like structures or OLCs from stem cells in the culture dish was observed.^52, 76, 80, 82, 83, 84, 85^ Particularly surprising is the formation of aggregates resembling cumulus-oocyte complexes from either female or male foetal porcine or newborn mouse SDSCs.^75, 86, 87, 88, 89, 90^ In all cases, however, the OLCs showed morphological and molecular abnormalities and were not proved to be functional. Our interpretation is that under certain *in vitro* conditions, some types of stem cells can acquire morphological and molecular features of oocytes independently from meiosis and proper follicle assembly.^91^ A recent paper supports such a possibility. In fact, Dokscin *et al.*^92^ showed that mouse oocyte growth and differentiation are dissociable from the chromosomal events of meiosis; they observed that a few surviving *Stra8*-deficient female germ cells grown and differentiated into OLCs that synthesized zona pellucida, assembled into follicles and were even ovulated and fertilized without meiotic initiation, and noteworthy, these oocytes arrested at the two-cell stage after *in vitro* fertilization.

Several papers reported the formation of haploid male germ cells *in vitro* resembling round spermatids or even with a sperm-like morphology from mouse^71, 82, 93, 94^ and human^73, 74, 95, 96, 97^ ES, iPS cells and adult stem cells. The efficiency of such processes was, however, very low and when tested, the haploid cells proved to be only partly functional. In fact, in the mouse, two papers showed that haploid cells generated *in vitro* were able to fertilize oocytes. Geijsen *et al.*^71^ detected and isolated haploid cells from mouse EBs treated with RA, and showed that the injection of the cells into oocytes resulted in the formation of blastocyst-like structures at a low rate. Nayernia *et al.*^93^ reported the induction of haploid cells with a sperm-like morphology from ES cell monolayers and successful production of offspring by intracytoplasmic injection which had growth abnormalities and died within 5 months after birth.

### Oocytes and gonocytes from *in vitro* cultured PGC-like cells inside gonadal tissues

As reported above, various experimental approaches indicate that it is relatively simple to induce the development of embryo-derived female PGCs into primary growing oocytes within *in vitro* cultured embryonic ovarian tissues. Under certain culture conditions, such oocytes are able to form follicles and in rare cases to complete the growing phase and be fertilized.^42^ In all cases, however, the OLCs inside these structures showed morphological and molecular abnormality and were not proved to be functional.

As far as we know, the only attempt to culture PGC-LCs *in vitro* within embryo-derived ovarian tissue has been reported by Hayashi and Surani.^98^ Stella-GFP-positive PGC-LCs obtained from EpiSCs were mixed with gonadal cells from 12.5-dpc ovaries, and after centrifugation, the cell suspension was left to aggregate and seeded through the gaps present between small pieces of 12.5-dpc ovaries on transwell inserts. After 40 days in culture, OLCs with strong expression of Stella-GFP indistinguishable from the endogenous germ cell present in the same co-cultures were observed although with very low frequency around 1 oocyte/5000 PGC-LCs.

The available information on male embryo-derived PGCs *in vitro* cultured within testis tissues apart for their ability to differentiate into prospermatogonia or gonocytes did not account for their capability to undergo spermatogenesis. Likely for this inability and the difficulties in reconstructing spermatogenesis *in vitro* were discussed in the previous paragraph; no attempts have been performed to maintain male PGC-LCs *in vitro* for a long time within testis tissues or onto Sertoli cell monolayers. In the works in which male PGC-LCs were identified and maintained in EBs up to the haploid status, we can postulate that such formation might develop into a testis-like structure in which PGC-LCs can differentiate at a certain extent.

The new methods allowing spermatogenesis in cultured postnatal or prepuberal gonocytes reported above represent a system to attempt to differentiate XY PGC-LCs to postnatal gonocytes/spermatogonia and even to accomplish complete *in vitro* spermatogenesis.

### Oocytes and sperm derived from *in vivo* transplanted PGC-LCs

Because as reported above, a number of papers showed that embryo-derived female PGCs, at least from 13.5 dpc onward, when aggregated with ovarian somatic cells and transplanted under the kidney capsule or ovarian bursa of syngeneic mice were able to give rise to fertilizable oocytes, some authors used this approach to investigate whether PGC-LCs possess such capability.

Nicholas *et al.*^80^ co-aggregated GFP-PGC-LCs obtained from mouse ES cells with dissociated newborn mouse ovarian tissues. After transplantation under the kidney capsule of recipient mice, the authors found some GFP oocytes within primary follicles in two of the grafts. Similar results were obtained by Dyce *et al.* and Chuang *et al.*^90, 99^ Hayashi *et al.* published the most efficient method to obtain oocytes after aggregation of GFP-PGC-LCs obtained from mouse EpiLCs (see above) with 12.5-dpc ovarian cells and *in vivo* transplantation under the ovarian bursa of host mice. These authors reported an efficiency of about 0.7% fully grown oocyte generated after 4 weeks in a synchronous single wave of oogenesis from both female PGC-LCs and embryo-derived PGCs.

Hayashi *et al.*^9^ also reported that male PGC-LCs from EpiLCs, like embryo-derived PGCs,^63^ were capable to differentiate into spermatozoa when transplanted into seminiferous tubules of prepuberal mice. Such spermatozoa gave rise to healthy individuals through in *vitro* microinsemination. Zhu *et al.*^100^ reported that male germ cells produced from mouse iPS cell EBs were able to differentiate into male germ cells ranging from spermatogonia to round spermatids after transplantation into recipient testes. Toyooka *et al.*^61^ should be credited for the first successful generation of spermatozoa from male PGC-LCs. The authors using BMP4 to induce germ cells from EBs, reported that male PGC-LCs were able to differentiate into spermatozoa in aggregates with gonadal cells from 12.5-dpc testis when transplanted under a host testis capsule. However, the capability of these cells to fertilize was not investigated.

## Conclusions

The artificial germ cells produced in the mouse proved to be functional because they were capable to differentiate into spermatozoa and oocytes that give rise to healthy individuals. In this regard, the behaviour of the mouse PGC-LCs was quite similar to that of embryo-derived PGCs. However, these still showed some notable differences from their *in vivo* counterparts. Although the reasons of such differences are not clear, these results indicate that PGC-LCs are not identical to embryo-derived PGCs and suggest that the gonadal ridges produce factor(s) essential for PGC survival/proliferation and differentiation. Therefore, it is not surprising that both artificial and real PGCs require to be enclosed inside or re-aggregated with suitable somatic cells to progress through gametogenesis. However, culture conditions suitable to complete oogenesis and spermatogenesis *in vitro* have not been established yet.

Generally, two processes that are the most difficult to be reconstructed in isolated PGC-LCs *in vitro* are the proper entry and progression into the meiosis and the epigenetic changes accompanying both female and male gametogenesis. Determining whether epigenesis and meiosis occur correctly during the process of deriving germ cells from stem cells is obviously crucial for the acquisition of proper gamete functionality. Nevertheless, the apparent failure of stem cell-derived germ cells to undergo meiosis efficiently could provide opportunities to study the molecular mechanisms regulating meiotic entry, progression and further broaden insights into the causes of meiotic failure.

As a prerequisite to the establishment of gametogenesis *in vitro*, we must first achieve a comprehensive understanding of the mechanisms underlying gametogenesis *in vivo*. Only the elucidation of the many still unknown aspects of such complex process and the progress in the coculture methods to maintain germ cells for long periods *in vitro* in 3-D microenvironments, suitable for the different stage-dependent requirements of gametogenesis, will give the necessary tools for rendering the production of germ cells from stem cells a reliable possibility. The first steps for producing germ cells from stem cells *in vitro* are promising, but there is still a long road ahead.

## Figures and Tables

**Table 1:** The role of key genes involved in germ cell formation

**Genes**	**Role in germ cell development**	**References**
*T (BRACHYURY)*	Essential for robust activation of *Blimp1* and *Prdm14*	Aramaki *et al.*^101^
*Blimp1 (PRDM1)*	Stabilize germline transcription and repress neuronal differentiation	Sasaki *et al.*^18, 19, 102^
*Prdm14*	Promote the expression of germ cell genes and repress somatic genes	Grabole *et al.*^20, 21^
*Tcfap2c (AP2γ)*	Mediating *Prdm1*-induced suppression of mesodermal differentiation	Weber *et al.*^103^
*Fragilis*	Promotes germ cell competence from their somatic neighbors	Saito *et al.*^104^
*Dazl*	Regulates the translation of specific transcripts important for germ cell formation	Reynolds *et al.*^105^
*Mvh (VASA)*	Required for progression through meiotic prophase I in male mice	Tanaka *et al.*^106^
*Stra8*	Essential for the initiation of meiosis	Anderson *et al.*^107^

## Abbreviations

ES: embryonic stem
PGCs: primordial germ cells
PGC-LC: PGC-like cells
OLCs: oocyte-like cells
iPS: induced pluripotent stem
dpc: days post coitum
dpp: days postpartum
BMP: bone morphogenetic proteins
WNT3: wingless-type 3
KL: kit ligand
RA: retinoic acid
EG: embryonic germ
GSK3: glycogen synthase kinase 3
ERK: extracellular signal regulated kinase
AGM: aorta-gonad-mesonephros
ActA: Activin A
SSCs: spermatogonial stem cell
GFP: green fluorescent protein
OSCs: oogonia stem cells
EBs: embryoid bodies
SDSCs: skin-derived stem cells
EpiLCs: epiblast-like cells, EpiSCs, epiblast stem cells
